# Humeral biepicondylar fracture dislocation in a child: A case report and review of the literature

**DOI:** 10.1186/1757-1626-1-163

**Published:** 2008-09-19

**Authors:** Naseem Ul Gani, Abdul Qayoom Rather, Bashir Ahmed Mir, Manzoor Ahmed Halwai, Mudassir Maqbool Wani

**Affiliations:** 1 Department of orthopedics, Govt hospital for bone and joint surgery Barzullah, Srinager, India; 2 Department of surgery, GMC, Srinagar, India

## Abstract

**Introduction:**

Humeral biepicondylar fracture dislocation is a very rare injury reported only once in English literature by G R Taylor et al. We report a case of humeral biepicondylar fracture dislocation in a 13-year-old girl with a unique mechanism of injury.

**Case presentation:**

A 13-year-old girl presented with trauma elbow. Radiographs showed biepicondylar fracture of humerus with dislocation of elbow.

**Conclusion:**

In humeral biepicondylar fracture dislocation, reduction is always unstable. So treatment is open reduction and internal fixation.

## Introduction

The elbow is a common site of injury in children [1]. Medial epicondyle fracture of humerus is third most common pediatric elbow fracture(behind supracondylar & lateral condyle fracture) occurring mostly in age group 9–14 years. Fracture of the medial epicondyle is usually caused by a valgus stress producing traction on flexor pronator tendon and subsequently on the medial epicondyle itself. The valgus stress may be produced by a fall on out stretched hand or by a fall on elbow [1]. In children avulsion of the medial epicondyle is commonly associated with posterolateral elbow dislocation due to tight attachment of the ulnar collateral ligament both to medial epicondyle and ulna. Avulsion of the lateral epicondyle is rare and may be overlooked due to its late and unusual pattern of ossification [2]. Fracture lateral epicondyle is usually caused by a severe varus force applied to the elbow or a sudden severe tension in the extensor group of forearm muscles or it may also result from a fall on the outstretched hand, driving the head of the radius against the capitellum and displacing it together with the epiphysis and part of metaphysis[3]. Humeral biepicondylar fracture dislocation is a very rare injury reported only once in English literature by G R Taylor et al [2]. We report a case of humeral biepicondylar fracture dislocation in a 13-year-old girl with a unique mechanism of injury.

## Case presentation

**Figure 1:**
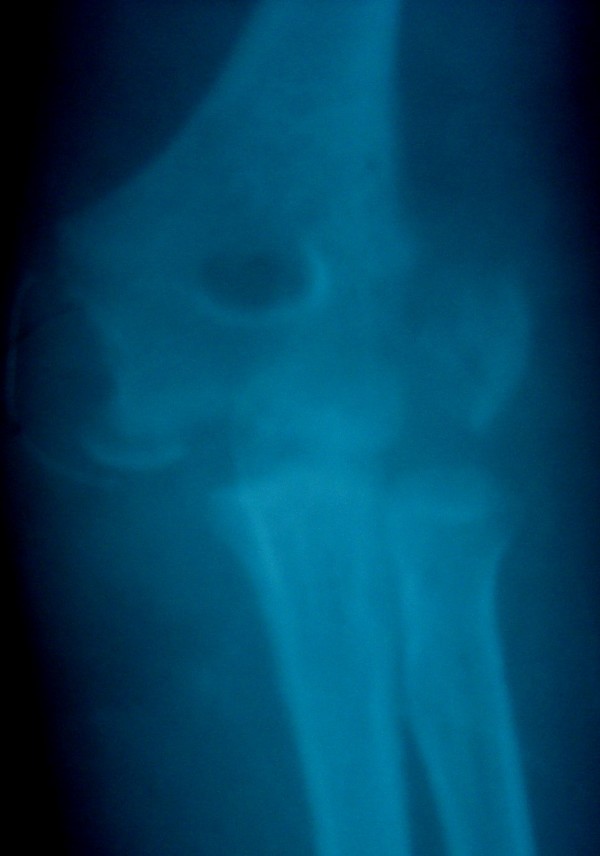
pre operative radiograph AP view.

**Figure 2:**
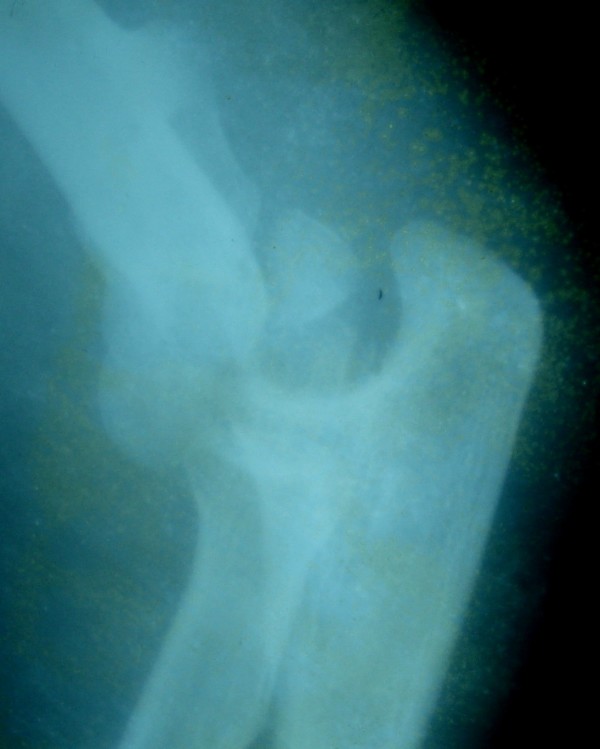
pre operative radiograph lateral view.

**Figure 3:**
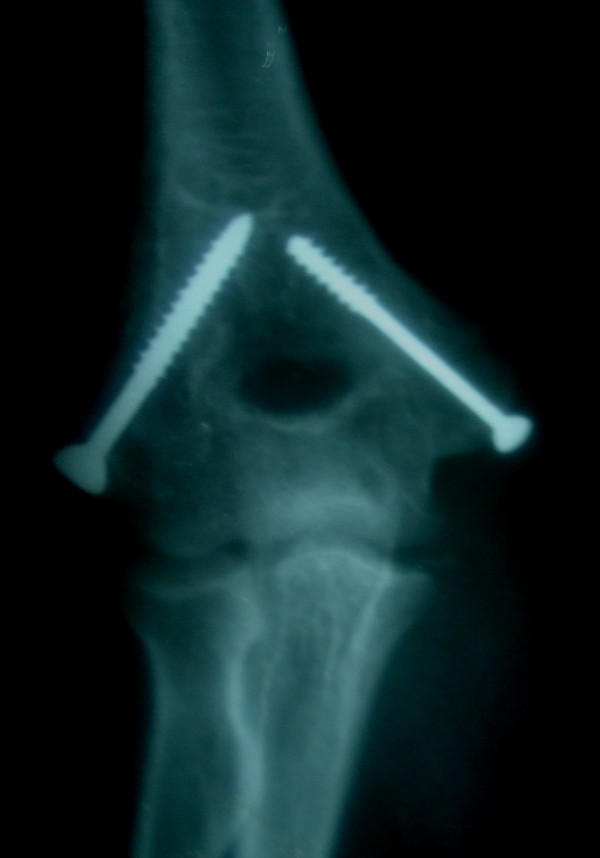
**post operative radiograph Ap view**.

A 13 year old girl while sitting on the floor with her right outstretched hand supporting the body sustained injury of right elbow when a Samawar(A traditional copper pot, 10–15 kg's in weight used for boiling tea and keeping tea hot using charcoal as fuel-) felt on her elbow. Patient presented within 5 hours of sustaining injury in emergency department of our hospital. Radiographs showed posterolateral dislocation of elbow with fracture of both medial and lateral epicondyle (Figure 1: AP view, Figure 2: Lat view). Distal neurovascular status was ok. Dislocation was closed reduced but the elbow was grossly unstable. Both fractures were open reduced and fixed with screws. Post operative radiographs were satisfactory (Figure 3: AP view,). Arm was splinted in a plaster slab for 6 weeks. After removing the slab, patient was advised range of motion exercise and patient regained full asymptomatic range of motion by 5 months.

## Discussion

There is limited evidence available in the literature describing complex humeral biepicondylar fracture dislocation in children. The complex elbow anatomy and multiple growth centers appearing at different time periods complicate the diagnosis and management of such injuries. Mechanism of injury in such a complex fracture dislocation remains to be resolved.

Lateral epicondylar fracture can result from a severe varus force applied to the elbow or due to severe sudden tension in the extensor muscles of forearm. A fracture may also result from a fall on outstretched hand, driving the radial head against capitellum and displacing it together with the epiphysis and part of the maphysis[3]. Fracture of the medial epicondyle is usually caused by the valgus stress producing traction on flexor pronator tendon. Valgus stress may be produced by a fall on the outstretched hand or by a fall on the elbow[1].

It is likely that in this child who was sitting with her right outstretched hand supporting her body, samawar fell on her extended elbow causing direct impact to the lateral epicondyle leading to its fracture and simultaneously producing a vulgus stress at elbow leading to fracture of medial epicondyle and dislocation of elbow.

Another possibility is that the samawar fell on extended elbow of the child who was sitting with her right outstretched hand supporting her body, producing a valgus stress at the elbow and simultaneously driving the radial head against the capitellum and fracturing it together with the lateral epicondyle.

It is also possible that in this child who was sitting with her right outstretched hand supporting her body, Samawar fell on her extended elbow producing a valgus stress at elbow and simultaneously causing internal rotation and medial displacement of humerus over the fixed hand leading to epiphysial traction injury on both sides.

This injury would account for gross instability of elbow. In our case open reduction and internal fixation of both fractures restored elbow stability. Patient had regained full range of movement by 5 months. Though there is very little literature available on this injury, we believe that closed reduction and conservative management will lead to a grossly unstable and easily dislocatable elbow. So we agree with Taylor [2], who recommended open anatomical reduction to ensure restoration of elbow stability. We also agree with Taylor [2] that this injury may be missed in a young child as the lateral epiphysis ossifies in the second decade, though this was not seen in our case as the child was of 13 years age.

## Conclusion

This injury would account for gross instability of elbow, so open reduction and internal fixation is recommended to restore elbow stability.

## Competing interests

The authors declare that they have no competing interests.

## Authors' contributions

NUG designed the study, wrote the manuscript, performed literature review, AQR, BAM, MAH, MMW helped in literature review and drafting the final manuscript. All authors read and approved the final manuscript.

## Consent

Written informed consent was obtained from the patient's guardian for publication of this case report and accompanying images. A copy of the written consent is available for review by the Editor-in-Chief of this journal.
